# Oxidative Stress and Mitophagy in Rats With Nitroglycerin/Inflammatory Soup−Induced Migraine: A Comparative Study

**DOI:** 10.1155/prm/5584799

**Published:** 2026-04-01

**Authors:** Wen-xiu Sun, Ting-yan Chen, Mao-mei Song, Ying-jie Gao, Sui-yi Xu

**Affiliations:** ^1^ Department of Neurology, Headache Center, The First Hospital of Shanxi Medical University, Taiyuan, 030001, Shanxi, China, sxmu.edu.cn; ^2^ Department of Neurology, Headache Center, Shenzhen Baoan People’s Hospital, The Second Affiliated Hospital of Shenzhen University, Shenzhen, 518000, Guangdong Province, China, szu.edu.cn

**Keywords:** antioxidant, mitochondrial metabolism, oxidative stress, rat migraine model, trigeminal nuclear caudalis

## Abstract

**Objective:**

Oxidative stress leads to mitochondrial DNA, protein, and lipid damage, ultimately resulting in mitochondrial dysfunction. Decreased mitophagy leads to the continuous accumulation of damaged mitochondria, which further induces oxidative stress and cellular damage. Mitochondrial dysfunction and an imbalance between energy supply and demand may lead to increased migraine susceptibility. However, there have been no comparative studies from the perspective of oxidative stress and mitophagy.

**Method:**

Male Sprague−Dawley rats were divided into control, NTG (10 mg/kg, intraperitoneal, Days 1/3/5/7), and IS (epidural infusion, 7 days) groups (*n* = 10, per group). Paw mechanical thresholds, migraine‐like behaviors (head–face scratching, body grooming, exploration, and freezing time), and oxidative markers (malondialdehyde, catalase, reduced glutathione, and superoxide dismutase) in the trigeminal nucleus caudalis were assessed. The expressions of PINK1, Parkin, LC3II/I, TOMM20, and COXIV were evaluated to quantify levels of mitophagy.

**Results:**

Both NTG/IS models reduced mechanical thresholds, but NTG induced stronger head scratching and freezing time. Oxidative stress markers diverged: NTG elevated malondialdehyde and depleted reduced glutathione/superoxide dismutase, while IS primarily reduced superoxide dismutase. Both models exhibited a decreased LC3II/I ratio as well as reduced PINK1 and Parkin.

**Conclusion:**

NTG/IS administration comparatively decreased the mechanical threshold, increased the level of oxidative stress, and decreased the level of mitophagy in the trigeminal nucleus caudalis in migraine rats.

## 1. Introduction

In recent years, energy metabolism has received increasing attention in the pathogenesis of migraines. Disorders of mitochondrial energy metabolism increase oxidative stress and impair antioxidant defense [1]. A total of 46.9% of patients with migraine show abnormally high peroxide levels and reduced activity of antioxidant enzymes [1, 2]. Catalase (CAT), reduced glutathione (GSH), and superoxide dismutase (SOD) play important roles in antioxidant defense [3]. CAT is the hallmark enzyme of the peroxisome that scavenges H_2_O_2_ produced during metabolism to prevent oxidative damage to cells. GSH is the most abundant sulfhydryl oxidant in mammalian cells and plays a key role in maintaining intracellular redox homeostasis [4]. SOD is an enzyme that catalyzes the generation of oxygen and peroxides from superoxide anion radicals, and as an endogenous antioxidant, it can play a role in repairing cells and reducing peroxide damage [5]. SOD_2_ is the only antioxidant enzyme in the mitochondria that catalyzes the conversion of reactive oxygen species to hydrogen peroxide. Mitochondria are energy factories, and respiratory chain dysfunction may be a factor in migraine attacks [6]. Mitochondrial dysfunction is associated with the susceptibility to migraines and their severity [7]. Migraine model studies in rats have revealed the presence of abnormal mitochondrial dynamics and impaired mitochondrial biogenesis [8]. Mitophagy cleans damaged mitochondria and reduces oxidative damage by phagocytosis and degradation of oxidizing substances [9]. Mitophagy can be categorized into polyclonal anti‐PTEN−induced kinase 1 (PINK1)/Parkin‐dependent and nondependent types. The PINK1/Parkin signaling pathway mediates mitophagy and binds to LC3 to degrade damaged mitochondria [10].

Nitroglycerin (NTG) and inflammatory soup (IS) activate oxidative stress in animal models, leading to migraine‐like attacks [11]. The NTG model is based on the vasogenic theory and presents a pathophysiological process closely resembling that of human migraine [12]. The model is simple, economical, and reproducible and has become a commonly employed animal model for migraine research. This feature may correspond to the clinical subset of migraine patients who present with pulsatile headache (suggesting a vascular origin) and respond favorably to vasoconstrictors such as triptans. High concentrations of nitric oxide substantially reduce mitochondrial respiratory chain activity, such as at the binding sites of cytochrome oxidase for oxygen and nicotinamide adenine dinucleotide dehydrogenase, leading to reduced adenosine triphosphate production, triggering migraine attacks [13]. Repeated meningeal nociception leading to chronic trigeminal oversensitization is an accepted animal migraine model [14]. The trigeminal vascular system consists of sensory neurons innervating the meninges and blood vessels, which conduct via the trigeminal nucleus caudalis (TNC) and project to the ventromedial thalamic nucleus [15]. This model may recapitulate migraine associated with neuroinflammation or dural mast cell degranulation. However, this model is not immune to the effects of surgical manipulation, and chemical irritants may alter the blood−brain barrier system. Studies have been conducted to mimic the pathological process of migraines by inducing brain energy metabolism disorders in NTG/IS rat models [8, 16]. However, there have been no comparative studies from the perspective of oxidative stress and mitophagy.

## 2. Materials and Methods

### 2.1. Experimental Design

All the experimental procedures were approved by the Ethics Committee of the Laboratory Animal Center of the First Hospital of Shanxi Medical University. Male Sprague−Dawley rats were provided by the Laboratory Animal Center of Shanxi Medical University. Thirty healthy male rats (6–8 weeks, weight: 200–250 g) were randomly divided into the control, NTG, and IS groups (*n* = 10). Rats were placed in an environment with a temperature of 23 C ± 2°C, humidity controlled at 50% ± 10%, a 12‐h light/dark cycle, and with free access to food and water. The rats were housed for 1 week to acclimatize them to the environment before starting the experiment.

### 2.2. NTG‐Induced Migraine Rat Model

Referring to the previous migraine model protocol in rats [17], 10 mg/kg of NTG was injected intraperitoneally every other day, for a total of four infusions on Days 1, 3, 5, and 7. Controls infusions were administered for the same number of experimental days.

### 2.3. IS‐Induced Migraine Rat Model

IS molding was induced by repeated meningeal nociception as previously reported [14]. Anesthesia was induced in the rats with 5% isoflurane using a compact small animal anesthesia machine (RWD, China), followed by maintenance with 1.5%–2.5% isoflurane using a face mask. The rats were placed on a brain stereotaxic apparatus (RWD, China) for routine skin preparation and sterilization. A median incision was made on the head, the subcutaneous tissues were separated bluntly, and the fontanel was exposed. A dental drill was used to create a cranial window with an aperture of approximately 1 mm in diameter, 1.5 mm posterior and 1.5 mm lateral to the fontanel. A microcannula was placed in the target cranial window, embedded, and fixed with dental cement. The rats were housed in separate cages and allowed to recover for 7 days. An epidural infusion of 10 μL IS (phosphate buffer containing 1 mM histamine, 5‐hydroxytryptamine, bradykinin, and 0.1 mM prostaglandin E2, pH = 7.4) was delivered through the microcannula. IS was administered once daily for 7 days.

### 2.4. Paw Mechanical Threshold (PMT) Measurement

The PMT was tested in the rats with 0.6–15 g of Von Frey filament (Touch‐Test, USA) [8, 18]. The rats were placed in cages with wire mesh bottoms for 3 days in advance to acclimate. Using the up‐and‐down method, the PMT was induced in rats by applying a Von Frey filament perpendicular to the central region of the paw. The 50% PMT was recorded in rats half an hour before and 2 h after drug administration [19]. The PMT was continuously monitored for 7 days. Each stimulus was separated by at least 30 s to ensure that the animals regained their quiet state before proceeding to the next stimulation session.

### 2.5. Behavioral Observation

Three days prior to drug administration, the rats were acclimated in Plexiglas cages for 30 min. Head–face scratching, body grooming, exploration, and freezing time were recorded within 1 h of NTG/IS administration [20]. The experiments were conducted daily at fixed times between 9:00 a.m. and 4:00 p.m. and recorded for 7 consecutive days. Scratching and grooming behaviors were characterized by continuous claw contact with the head and body. Exploratory behaviors included running, climbing, and sniffing. Freezing behavior was manifested by maintaining a fixed position with the limbs touching the ground, with the whiskers immobile.

### 2.6. Oxidative Stress Indicator Test

The rats were deeply anesthetized and sacrificed by decapitation 24 h after modeling. TNC samples were collected to detect oxidative stress−related indicators, including SOD, MDA, GSH, and CAT (Njjcbio, China). The assays included the following: SOD activity was determined by the xanthine oxidase method, in which superoxide anion radicals were generated by the reaction between xanthine and oxidase. The latter oxidized hydroxylamine to produce nitrite, as detected by the absorbance [21]. Lipid peroxidation was assessed by measuring the MDA concentration using a method based on spectrophotometric analysis of the reaction of thiobarbituric acid (TBA) with MDA [22]. The MDA concentration was calculated by measuring the absorbance of TBA‐reactive substances at 532 nm. GSH reacts with 5,5′‐dithio‐2‐nitrobenzoic acid to form a yellow compound that colorimetrically quantified at 405 nm [23]. CAT activity was measured by analyzing the rate at which it caused the decomposition of H_2_O_2_ at 405 nm, the substrate of the enzyme contained in various tissue samples [24].

### 2.7. Immunofluorescence Assay

On the day after modeling, the rats were anesthetized and their hearts exposed. The brain tissue was isolated and prepared for dissection by perfusion with precooled saline until the blood was flushed out, followed by perfusion fixation with 4% paraformaldehyde. Brain tissues were dehydrated with sucrose, embedded at the optimal cutting temperature, and placed in a frozen sectioning machine (Leica, Germany). The TNC was serially sliced to a thickness of 20 μm. After antigen repair, membrane breaking, and blocking, the primary antibody was incubated overnight at 4°C, and the secondary antibody was incubated for 2 h at 23 C ± 2°C. After antigen retrieval, permeabilization, and blocking, the samples were incubated overnight at 4°C with primary antibodies (rabbit polyclonal anti‐LC3II/I, 1:500, Abcam, UK; mouse monoclonal anti‐translocase of outer mitochondrial membrane 20 [TOMM20], 1:100, Santa Cruz Biotechnology, USA). Subsequently, the samples were incubated at room temperature for 2 h with secondary antibodies (Alexa Fluor 488−labeled goat anti‐mouse IgG [H + L], 1:500; Alexa Fluor 555−labeled donkey anti‐rabbit IgG [H + L], 1:500, Beyotime, China). The sections were stained with 4′,6‐diamidino‐2‐phenylindole and mounted on a fluorescence microscope (10x magnification). Three consecutive slices of the TNC region were isolated for each rat following “The Rat Brain In Stereotaxic Coordinates” [25].

### 2.8. Western Blot

The target protein concentration was determined according to the instructions provided by the bicinchoninic acid (BCA) Protein Concentration Assay Kit (Boster, China). An aliquot of the leveled protein sample was separated on a 12% sodium dodecyl sulfate−polyacrylamide gel with the initial voltage set at 80 V, which was then increased to 120 V as bromophenol blue entered the separation gel. The electrophoresis duration was 90 min. Next, the membrane was transferred using a semidry membrane transfer device (Bio‐Rad, USA) at 16 V for 25 min. After the transfer, the protein membranes were blocked for 2 h at room temperature. The following antibodies were diluted using a western blot−specific antibody dilution buffer (Boster, China): Rabbit PINK1 (1:5000, Abcam, UK), rabbit polyclonal anti‐Parkin (1:2000, CST, USA), Rabbit polyclonal anti‐light chain‐3 protein II/I (LC3II/I) (1:5000, Abcam), Rabbit polyclonal anti‐TOMM20 (1:5000, Boster), Rabbit polyclonal anti‐cytochrome c oxidase IV (COXIV) (1:5000, CST, USA), rabbit polyclonal anti‐glyceraldehyde‐3‐phosphate dehydrogenase (GAPDH) (1:5000, Abclonal), and goat anti‐rabbit immunoglobulin G horseradish peroxidase (IgG‐HRP) (1:5000, Abclonal). The secondary antibody was incubated for 2 h at 23°C ± 2°C, and antibody binding was detected using an Ultrasensitive Luminescent Substrate Kit (Boster, China). Signals were detected using a protein gel imaging system (Bio‐Rad, USA). ImageJ software (National Institutes of Health, Bethesda, MD, USA) was used to analyze the grayscale values of the target proteins, and the relative protein levels were calculated using GAPDH as an internal reference.

### 2.9. Statistical Analysis

Quantitative data are reported as means ± standard error (SE). Statistical analyses were performed using SPSS software (Version 26.0; SPSS Inc., Chicago, IL, USA). Statistical diagrams were generated using GraphPad Prism 8 software (GraphPad Software, San Diego, CA, USA). Two‐way analysis of variance (ANOVA) was used to compare the PMT and behavioral data between the control and NTG/IS groups. Other variables were analyzed using one‐way ANOVA, supplemented by the least significant difference *t*‐test. Statistical significance was set at *p* < 0.05.

## 3. Results

### 3.1. NTG/IS Administration Induces a Decrease in PMT in Rats With Migraine

**FIGURE 1:**
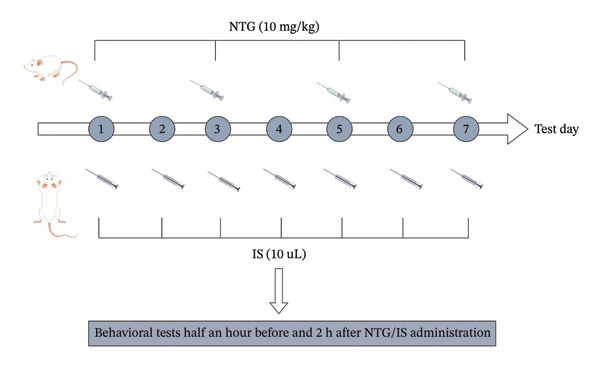
NTG/IS migraine rat modeling. PMTs were recorded 30 min before and 2 h after each administration. NTG group: intraperitoneal injection of NTG every other day (Days 1, 3, 5, and 7). IS group: IS administration via a microcatheter for 7 consecutive days. PMTs were recorded 30 min before and 2 h after administration. Abbreviations: NTG: nitroglycerin; IS: inflammatory soup; PMT: paw mechanical threshold.

Thresholds for the paw withdrawal response in rats (*n* = 7) were recorded 30 min before and 2 h after each drug administration (Figure 1). The basal PMT of the rats before and after repeated NTG/IS administration showed a decreasing trend (Figures 2(a) and 2(b)). The PMT of rats in both the NTG/IS groups dropped significantly to below 2 g before and after the fifth day of drug administration (Figures 2(c) and 2(d)). The NTG/IS group was significantly different from the control group (*p* < 0.01).

FIGURE 2Repeated NTG/IS administration leads to reduced PMT in rats (*n* = 7). (a and c) Basal PMT measured before NTG/IS administration in rats. (b and d) PMT measured after NTG/IS administration. Comparison of the control and NTG groups: ^∗^ *p* < 0.05, ^∗∗^ *p* < 0.01, and ^∗∗∗^ *p* < 0.001. Comparison of the control and IS groups: ^#^ *p* < 0.05, ^##^ *p* < 0.01, and ^###^ *p* < 0.001. Abbreviations: NTG: nitroglycerin; IS: inflammatory soup; PMT: paw mechanical threshold.

**Figure:**
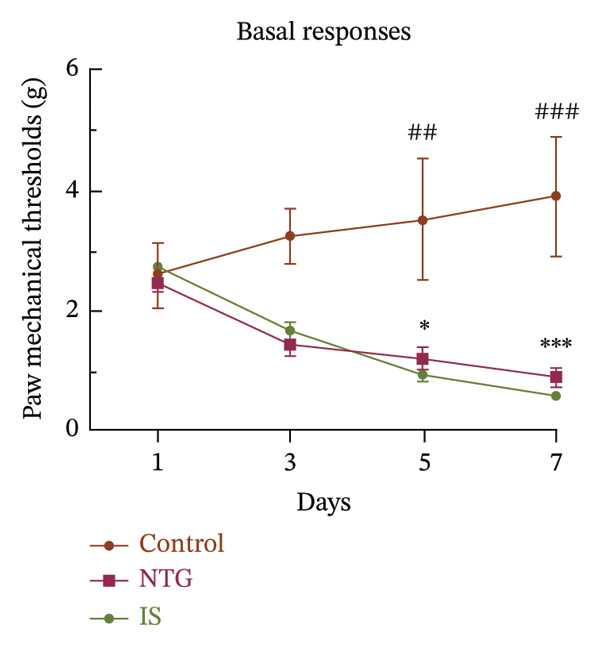
(a)

**Figure:**
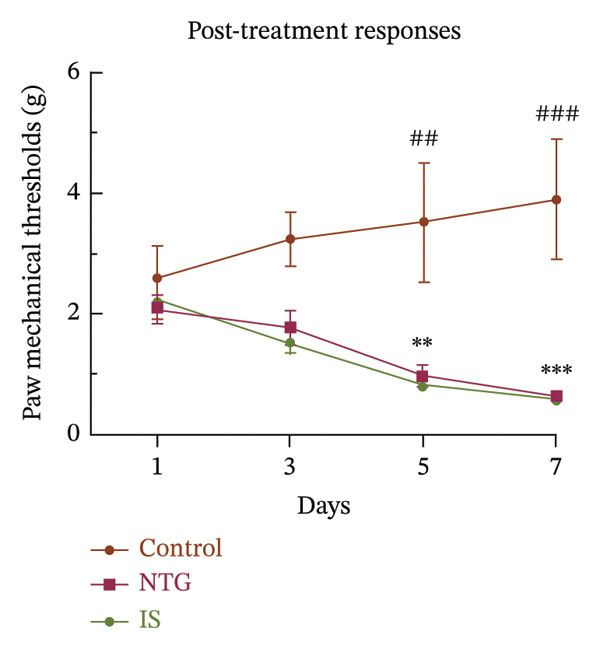
(b)

**Figure:**
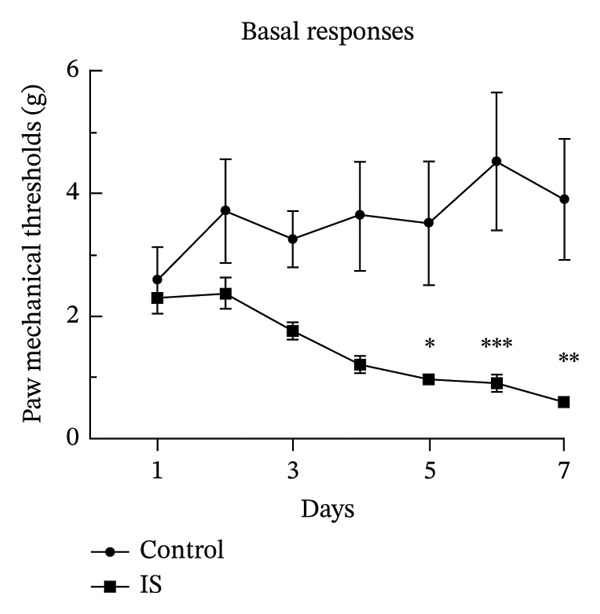
(c)

**Figure:**
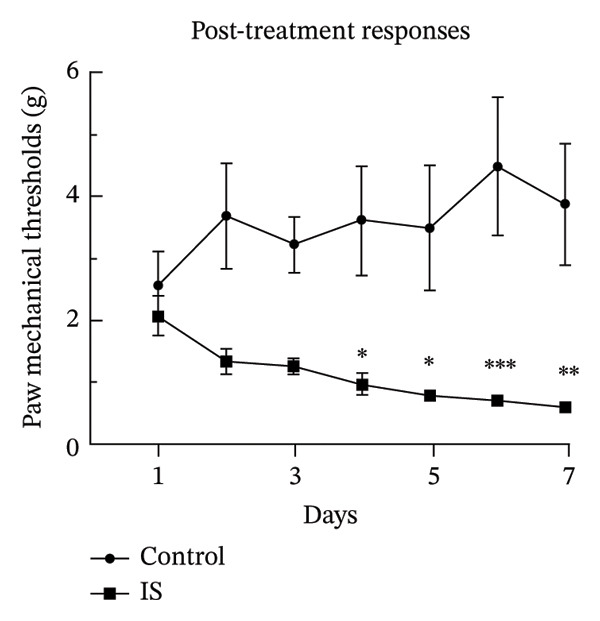
(d)

### 3.2. Rats Display Migraine‐Like Behavior After NTG/IS Administration

Head scratching, exploration, face and body grooming times, and freezing times were monitored within 1 h of administration (*n* = 10). Consistent with previous reports [20], rats exhibited significant migraine‐like symptoms after NTG/IS administration. The increase in head‐scratching time was more pronounced in the NTG group than in the IS group (*p* < 0.001) (Figure 3(a)). The NTG group showed significantly higher scratching time (*p* < 0.001) and freezing time (*p* < 0.05) on the third day of administration than control rats (Figures 3(a) and 3(d)). Face and body grooming and exploration times were significantly lower (*p* < 0.01) (Figures 3(b) and 3(c)). A decrease was observed in exploration time (*p* < 0.001), an increase in freezing time (*p* < 0.05), and a significant decrease in face and body grooming time on Day 7 (*p* < 0.05).

FIGURE 3Rats treated with NTG/IS presented migraine‐like behaviors on Days 1, 3, 5, and 7 (*n* = 10). (a) Head‐scratching behavior. (b) Exploration behavior. (c) Facial and body grooming behavior. (d) Freezing behavior. ^∗^ *p* < 0.05, ^∗∗^ *p* < 0.01, and ^∗∗∗^ *p* < 0.001. Abbreviations: NTG: nitroglycerin; IS: inflammatory soup.

**Figure:**
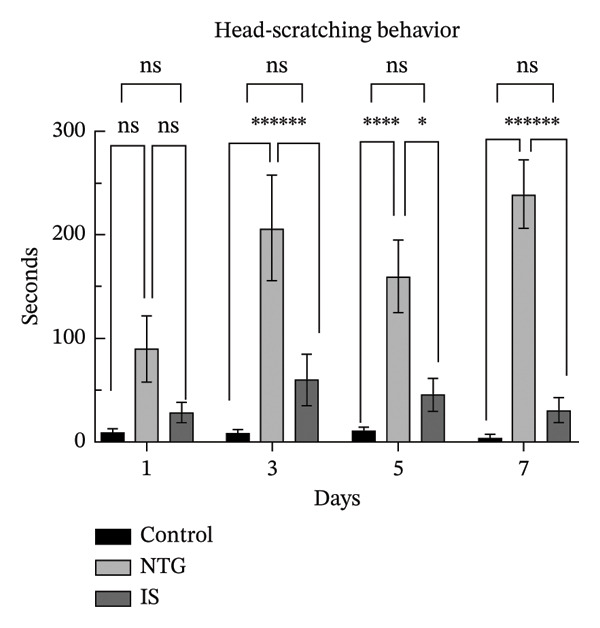
(a)

**Figure:**
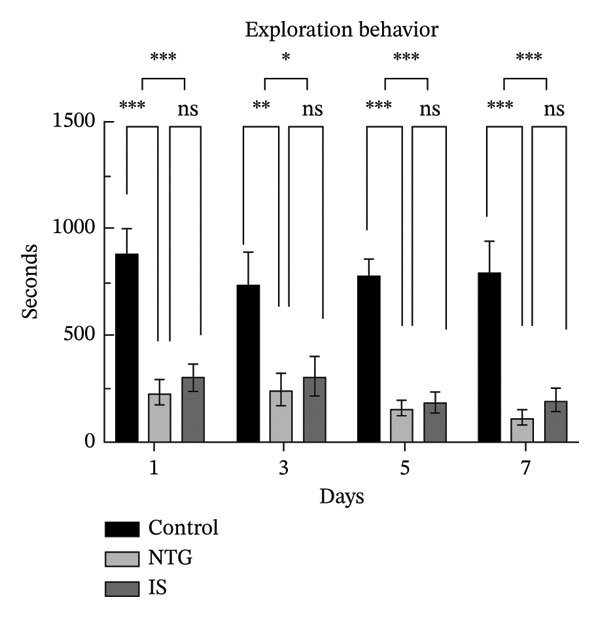
(b)

**Figure:**
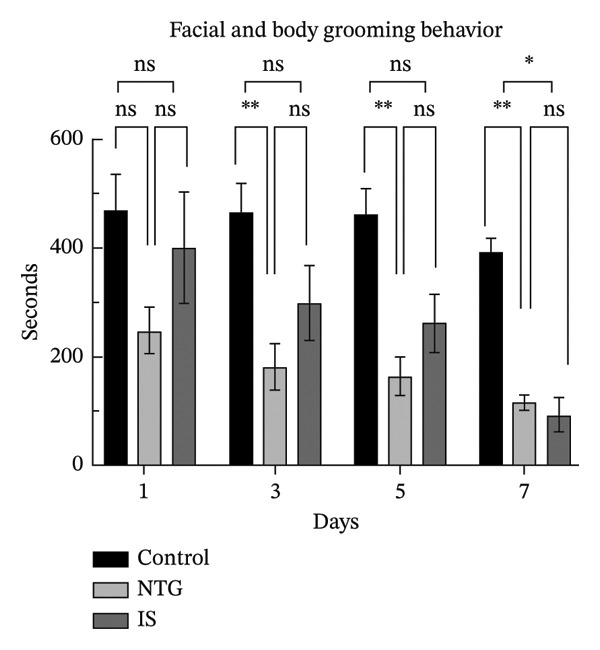
(c)

**Figure:**
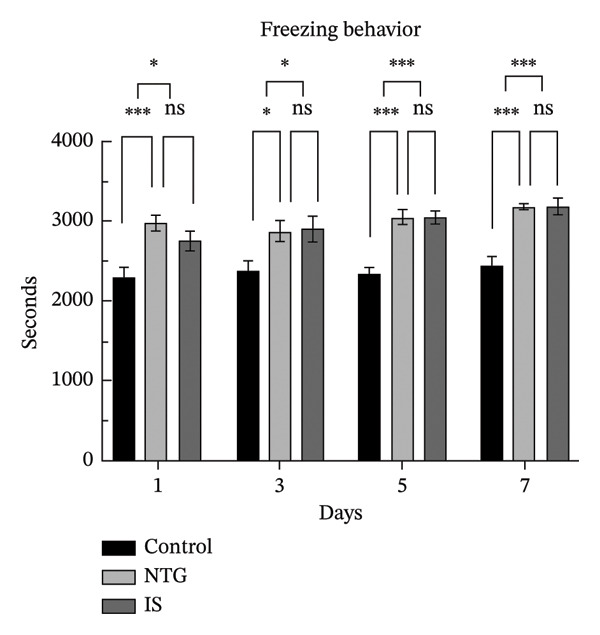
(d)

### 3.3. NTG/IS Administration Increases Oxidative Stress Levels at the TNC

As shown in Figure 4(c), the decreasing trend in GSH levels was more pronounced in the NTG group than in the IS group (*n* = 5, *p* < 0.01). MDA, a marker of lipid peroxidation, increased after NTG injection (*n* = 5, *p* < 0.05) (Figure 4(a)). In contrast to the trend of elevated MDA, the levels of GSH and SOD significantly decreased (*n* = 5, *p* < 0.001), and the content of CAT also decreased (*n* = 5, *p* < 0.05) (Figures 4(b), 4(c), and 4(d)). Compared to the control group, no statistical difference was noted in MDA levels (*n* = 5, *p* > 0.05), GSH and CAT levels were reduced (*n* = 5, *p* < 0.05), and SOD levels were significantly decreased (*n* = 5, *p* < 0.001) in the IS group. These results suggest that administration of both NTG/IS resulted in increased oxidative stress levels at the TNC in rats with migraine.

FIGURE 4Lipid peroxides and antioxidant capacity in the trigeminal nucleus caudalis of rats treated with NTG/IS (*n* = 5). (a) Malondialdehyde (MDA). (b) Catalase (CAT). (c) Reduced glutathione (GSH). (d) Superoxide dismutase (SOD). ^∗^ *p* < 0.05, ^∗∗^ *p* < 0.01, and ^∗∗∗^ *p* < 0.001. Abbreviations: NTG: nitroglycerin; IS: inflammatory soup; PMT: paw mechanical threshold; MDA: malondialdehyde; CAT: catalase; GSH: reduced glutathione; SOD: superoxide dismutase.

**Figure:**
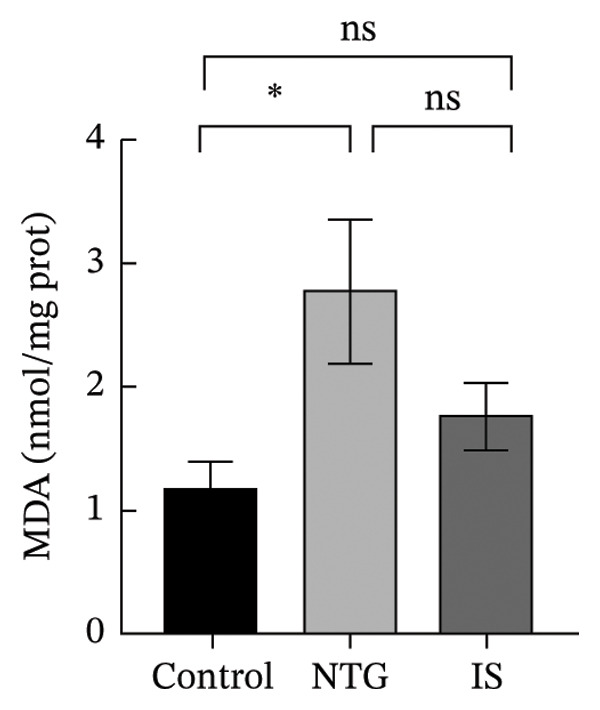
(a)

**Figure:**
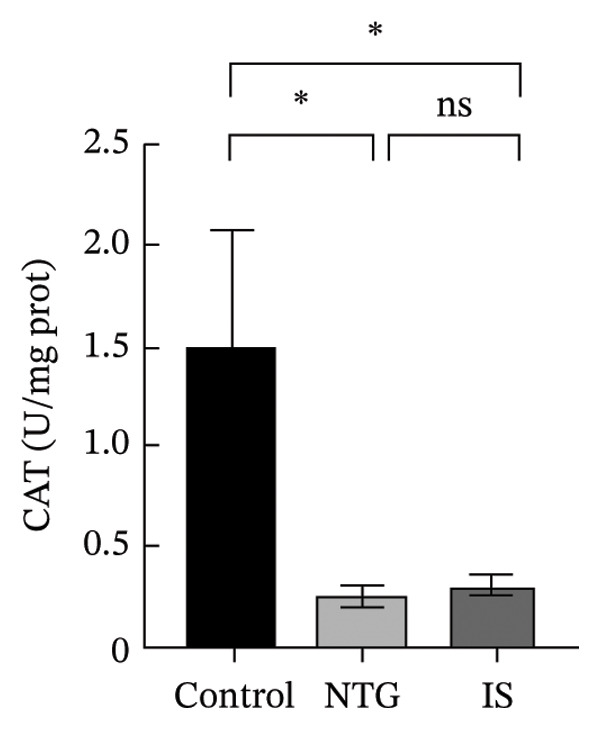
(b)

**Figure:**
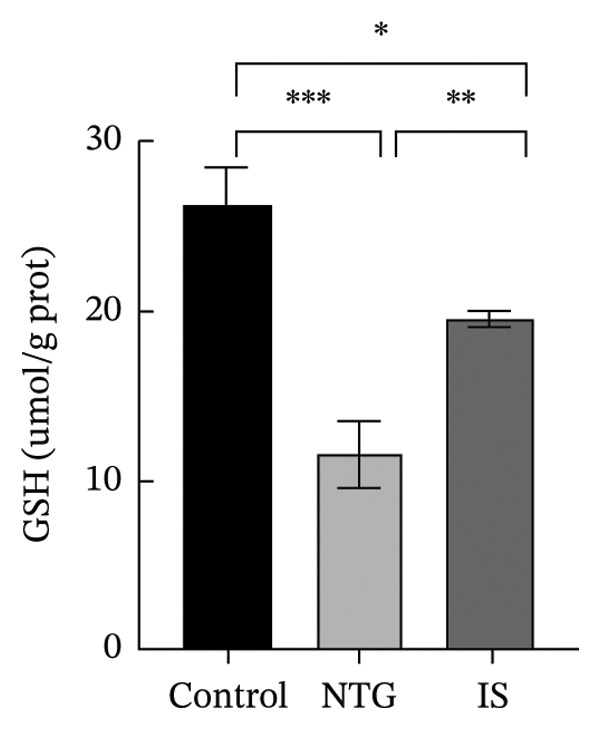
(c)

**Figure:**
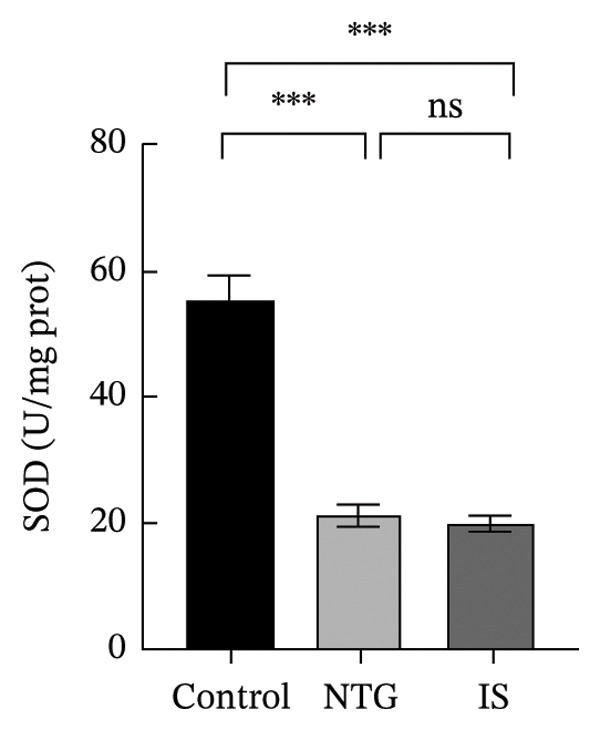
(d)

### 3.4. NTG/IS Administration Decreases Mitophagy Levels at TNC

**FIGURE 5:**
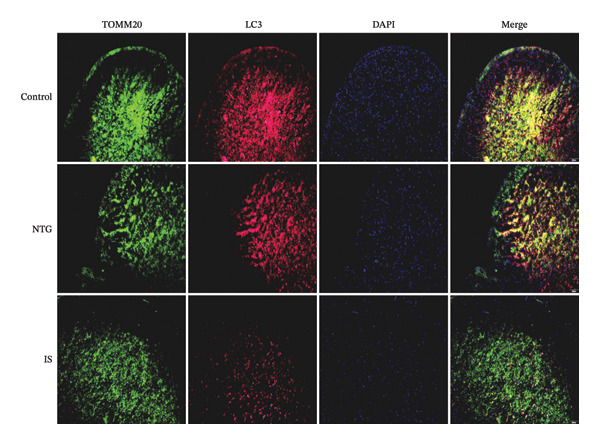
Double immunofluorescence staining with LC3 (red) and TOMM20 (green) in the trigeminal nucleus caudalis of NTG/IS rats (magnification 10x). Abbreviations: NTG: nitroglycerin; IS: inflammatory soup.

Double immunofluorescence staining of mitochondrial outer membrane proteins (TOMM20) and inner membrane proteins (COXIV) suggested that mitophagy levels may be decreased in the TNC in the NTG/IS migraine rat model (Figure 5). Western blot quantitative analysis (*n* = 3) revealed that the expression levels of PINK1, Parkin, and LC3II/I in NTG rats were significantly decreased compared to those in the control rats (*p* < 0.01) (Figures 6(b), 6(d), and 6(e)), whereas no significant difference was noted in elevation of TOMM20 (*p* > 0.05) (Figure 6(a)). COXIV expression levels in the NTG and IS groups were consistent with the TOMM20 trend, and no significant difference was observed (*p* > 0.05) (Figure 6(c)). These results suggest that the migraine rat model displayed decreased levels of mitophagy, presumably due to dysfunction of the autophagic degradation pathway.

FIGURE 6Mitophagy levels were decreased in the trigeminal nucleus caudalis of NTG/IS rats (*n* = 3). (a) TOMM20 expression. (b) LC3II/I expression. (c) COXIV expression. (d) PINK1 expression. (e) Parkin expression. ^∗^ *p* < 0.05 and ^∗∗^ *p* < 0.01. Abbreviations: NTG: nitroglycerin; IS: inflammatory soup.

**Figure:**
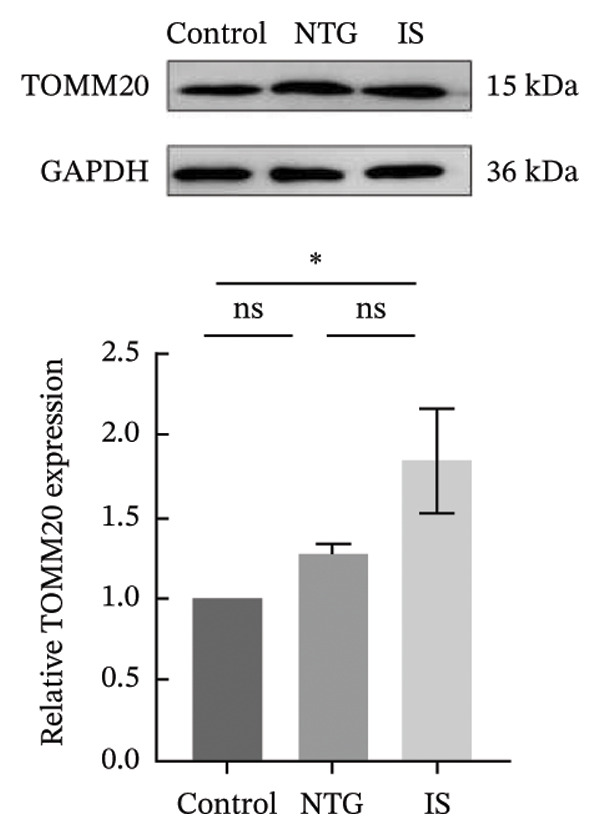
(a)

**Figure:**
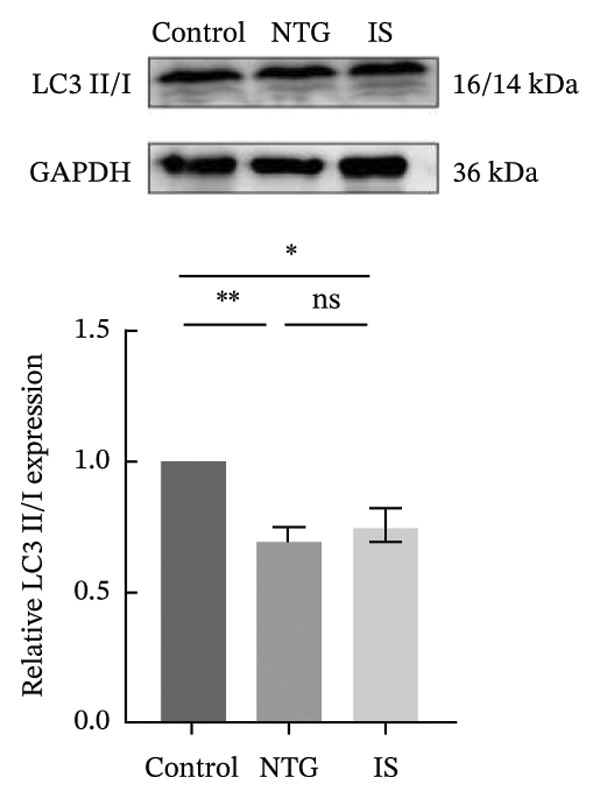
(b)

**Figure:**
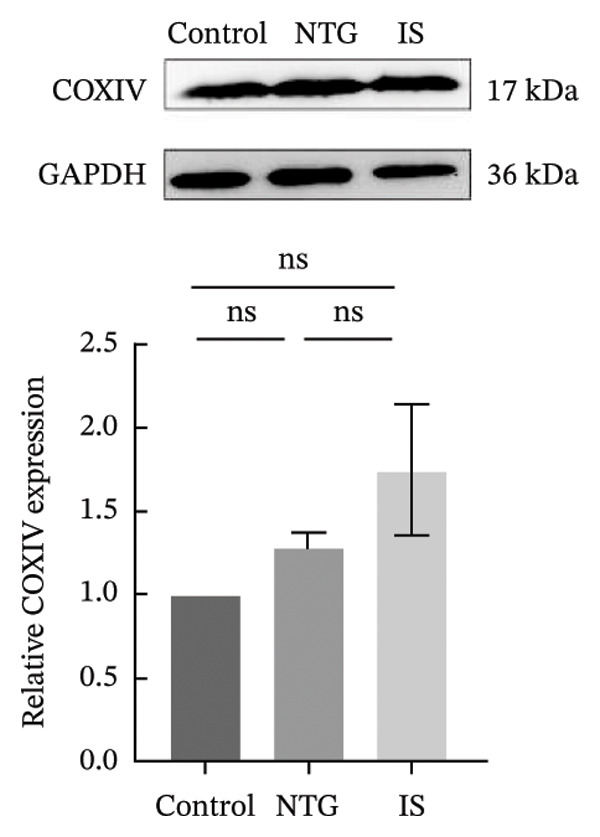
(c)

**Figure:**
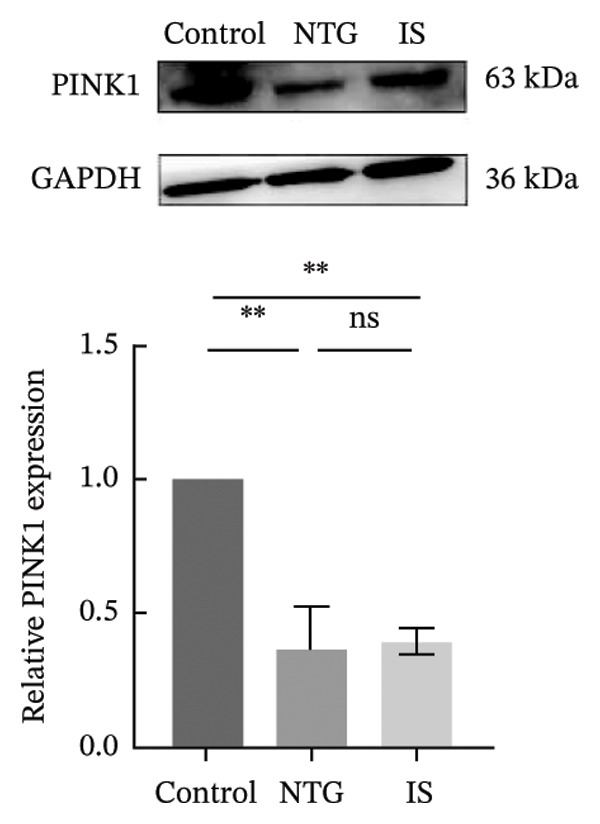
(d)

**Figure:**
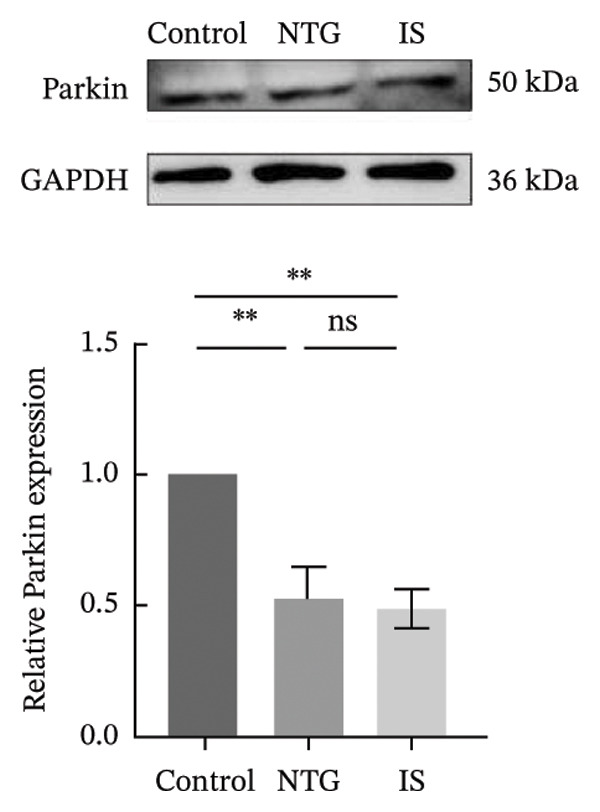
(e)

## 4. Discussion

Cutaneous pain in migraine is thought to result from the activation and sensitization of neurons in the trigeminal vascular pathway [26]. We found that head‐scratching behavior was more pronounced in the NTG model than in the IS model, suggesting that the behavioral assessment of different migraine models is variable. Animal experiments should be conducted using different migraine models depending on the purpose of the study. Oxidative stress leads to mitochondrial DNA, protein, and lipid damage, ultimately resulting in mitochondrial dysfunction [27]. Excessive oxidative stress can directly impair mitophagy. Oxidative modification of critical cysteine residues within the Parkin protein compromises E3 ubiquitin ligase activity, while oxidative damage to mitochondrial membrane proteins may disrupt PINK1 accumulation signaling [28]. Mitochondrial dysfunction and an imbalance between energy supply and demand may lead to increased migraine susceptibility [29]. In our study, the NTG/IS group showed elevated levels of oxidative stress compared to the control group, which is consistent with the results of a previous study.

Mitochondrial quality control is a finely tuned system that induces changes in mitochondrial quality and quantity [30]. Both the NTG and IS groups showed undifferentiated decreases in mitophagy. Decreased mitophagy leads to the continuous accumulation of damaged mitochondria, which further induces oxidative stress and cellular damage [31]. On the surface of damaged mitochondria, PINK1 accumulates and serves as an input signal for a positive feedback loop that recruits Parkin, which in turn facilitates mitochondrial degradation via mitophagy [32]. Our observation of decreased PINK1 and Parkin expression coinciding with elevated oxidative stress markers aligns with this notion. Mitochondrial dysfunction may result in abnormal calcium ion concentrations, excessive free radical production, decreased mitochondrial membrane potential, and imbalances in the opening and closing of the mitochondrial permeability transition pore [33]. We propose that reduced mitophagy contributes to neuronal hyperexcitability in the TNC through the following interrelated mechanisms: (1) Energy metabolic dysfunction: Impaired clearance of damaged mitochondria reduces ATP production, compromising Na^+^/K^+^‐ATPase activity. This destabilizes the neuronal membrane potential, thereby lowering the threshold for action potential generation. (2) Sustained ROS generation: Accumulated dysfunctional mitochondria serve as a persistent source of ROS, which can directly modulate voltage‐gated ion channels and neurotransmitter release. (3) Calcium dysregulation: Mitochondria with compromised membrane potential exhibit reduced calcium buffering capacity, leading to aberrant intracellular calcium transients that promote neuronal hyperexcitability. These changes may exacerbate neuronal energy depletion and apoptosis, thereby lowering the pain threshold and contributing to migraine. Similarly, clinical studies have confirmed the presence of markers of inflammation and oxidative stress in the plasma of patients with migraine during attacks, including increased levels of peroxides [34–36]. In addition, impaired energy metabolism in patients with migraines has been validated by imaging. Abnormalities in energy metabolism and metabolic changes in the dorsal pons of patients with migraine were revealed by positron emission tomography (PET), as well as an increased neuronal activation, rest glucose uptake ratio in the optic cortex on 18F‐fluorodeoxyglucose PET scans [37].

Fumaric acid esters exert protective effects against central sensitization by modulating endogenous antioxidant defense systems in an NTG mouse model [38]. Impaired astrocyte mitochondrial function [39] and dysregulation of the mitochondrial dynamic regulatory network in the trigeminal ganglion [8] have been observed in rat and mouse migraine models. Mitochondrial respiratory capacity decreased in the TNC of the rats in the IS model according to the Seahorse XF Analyzer [40]. Whether mitophagy levels are increased or decreased in migraine has been inconsistent. A study reported that the IS migraine rat model showed decreased autophagy and reduced LC3 levels, which is consistent with our findings [41]. However, Jiang et al. constructed a migraine mouse model using repeated intraperitoneal NTG injections and found that autophagic flux was impaired in the TNC, whereas LC3‐II levels were increased, suggesting an increase in mitophagy, contrary to our results. [16]. Several factors may account for this discrepancy: (1) Elevated LC3‐II levels can indicate either enhanced autophagosome formation or impaired lysosomal clearance. In our study, decreased LC3‐II, accompanied by reduced PINK1/Parkin expression, suggests impaired initiation of mitophagy. (2) Rats and mice exhibit distinct sensitivity and metabolic profiles in response to NTG. (3) Our intermittent dosing regimen allowed partial recovery between injections, which may have induced adaptive responses distinct from those resulting from daily cumulative exposure. We hypothesize that mitophagy is a double‐edged sword: moderate mitophagy removes senescent and damaged mitochondria, whereas excessive mitophagy leads to a decrease in the mitochondrial population, which affects energy metabolism.

This study has some limitations. First, although NTG/IS rats are currently the most widely used animal model of migraine worldwide and can mimic human migraine to a certain extent, they essentially experience drug‐induced secondary migraine−like attacks. Second, male rats were used in this study to avoid disturbances in the estrogen cycle. This limits our understanding of the role of estrogen in migraines. Third, the absence of a sham‐operated group in this study precludes definitive dissociation of the effects attributable to surgical manipulation and repeated isoflurane anesthesia from those specifically induced by inflammatory stimulation. Consequently, it remains possible that certain alterations observed in the IS group reflect, at least in part, a response to the surgical procedure rather than being exclusive to IS exposure. Of note, oxidative stress and mitophagy are dynamic processes that likely evolve over time during both the induction and remission phases of migraine. Our measurements may have captured only a transient snapshot of these events. Oxidative stress markers may fluctuate rapidly in response to trigeminovascular activation, while mitophagic flux may change dynamically over time as an adaptive response.

## 5. Conclusion

NTG/IS administration induces a decrease in mechanical thresholds in migraine rats, increases oxidative stress levels, and decreases mitophagy levels in the TNC. Considering the different routes of administration and induction mechanisms between the NTG and IS models, it is still necessary to explore more suitable animal models to study the role of oxidative stress and mitochondrial quality control in prophylactic migraine treatment.

**Nomenclature**
NTG: Nitroglycerin
IS: Inflammatory soup
TNC: Trigeminal nuclear caudalis
SOD: Superoxide dismutase
MDA: Malondialdehyde
GSH: Reduced glutathione
CAT: Catalase
ANOVA: Analysis of variance
SE: Standard error
PET: Positron emission tomography
BCA assay: Bicinchoninic acid assay

## Author Contributions

Conceptualization: Sui‐yi Xu. Data curation: Wen‐xiu Sun, Ting‐yan Chen, Mao‐mei Song, and Ying‐jie Gao. Formal analysis: Wen‐xiu Sun and Ting‐yan Chen. Investigation: Sui‐yi Xu. Methodology: Wen‐xiu Sun, Ting‐yan Chen, Mao‐mei Song, and Ying‐jie Gao. Project administration: Sui‐yi Xu. Resources: Sui‐yi Xu. Supervision: Sui‐yi Xu. Writing−original draft: Wen‐xiu Sun and Sui‐yi Xu. Writing−review and editing: Wen‐xiu Sun and Sui‐yi Xu.

## Funding

The study was funded by the Shanxi Basic Research Program (202303021221222).

## Disclosure

All authors approved the final version of the manuscript.

## Ethics Statement

All the experimental procedures were approved by the Ethics Committee of the Laboratory Animal Center of the First Hospital of Shanxi Medical University (SYXK 2024‐0008).

## Conflicts of Interest

The authors declare no conflicts of interest.

## Data Availability

The data that support the findings of this study are available from the corresponding author upon reasonable request.

## References

[1] Borkum J. M. , Brain Energy Deficit as a Source of Oxidative Stress in Migraine: A Molecular Basis for Migraine Susceptibility, Neurochemical Research, 2021, Springer, Berlin, Germany, 1913–1932. 10.1007/s11064-021-03335-9 33939061

[2] Gross E. C. , Putananickal N. , Orsini A. L. et al., Mitochondrial Function and Oxidative Stress Markers in Higher-Frequency Episodic Migraine, Scientific Reports. (2021) 11, no. 1, 10.1038/s41598-021-84102-2. PMC7907128 33633187

[3] Ighodaro O. M. and Akinloye O. A. , First Line Defence Antioxidants-Superoxide Dismutase (SOD), Catalase (CAT) and Glutathione Peroxidase (GPX): Their Fundamental Role in the Entire Antioxidant Defence Grid, Alexandria Journal of Medicine. (2018) 54, no. 4, 287–293, 10.1016/j.ajme.2017.09.001.

[4] Li Y. , Zhang Q. , Qi D. et al., Valproate Ameliorates Nitroglycerin-Induced Migraine in Trigeminal Nucleus Caudalis in Rats Through Inhibition of NF-кB, Journal of Headache and Pain. (2016) 17, no. 1, 10.1186/s10194-016-0631-z, 2-s2.0-84966412875. PMC4859223 27150105

[5] Bhat A. H. , Oxidative Stress, Mitochondrial Dysfunction and Neurodegenerative Diseases; A Mechanistic Insight, Biomedicine and Pharmacotherapy, 2015, Elsevier Masson, Amsterdam, the Netherlands, 101–110. 10.1016/j.biopha.2015.07.025 26349970

[6] Tiehuis L. H. , Koene S. , Saris C. G. , and Janssen M. C. , Mitochondrial Migraine; A Prevalence, Impact and Treatment Efficacy Cohort Study, Mitochondrion. (2020) 53, 128–132, 10.1016/j.mito.2020.05.004. 32464279

[7] Yorns W. R. and Hardison H. H. , Mitochondrial Dysfunction in Migraine, Seminars in Pediatric Neurology. (2013) 20, no. 3, 188–193, 10.1016/j.spen.2013.09.002, 2-s2.0-84890225838. 24331360

[8] Dong X. , Guan X. , Chen K. et al., Abnormal Mitochondrial Dynamics and Impaired Mitochondrial Biogenesis in Trigeminal Ganglion Neurons in a Rat Model of Migraine, Neuroscience Letters. (2017) 636, 127–133, 10.1016/j.neulet.2016.10.054, 2-s2.0-84999885125. 27984195

[9] Li D. , Ding Z. , Du K. , Ye X. , and Cheng S. , Reactive Oxygen Species as a Link Between Antioxidant Pathways and Autophagy, Oxidative Medicine and Cellular Longevity. (2021) 2021, no. 1, 10.1155/2021/5583215. PMC8324391 34336103

[10] Li J. , Yang D. , Li Z. et al., PINK1/Parkin-Mediated Mitophagy in Neurodegenerative Diseases, Ageing Research Reviews. (2023) 84, 10.1016/j.arr.2022.101817. 36503124

[11] Latif K. , Khan A. , Izhar Ul Haque M. , and Naeem K. , Bergapten Attenuates Nitroglycerin-Induced Migraine Headaches Through Inhibition of Oxidative Stress and Inflammatory Mediators, ACS Chemical Neuroscience. (2021) 12, no. 18, 3303–3313, 10.1021/acschemneuro.1c00146. 34455773

[12] Sureda-Gibert P. , Romero-Reyes M. , and Akerman S. , Nitroglycerin as a Model of Migraine: Clinical and Preclinical Review, Neurobiology of pain. (2022) 12, 10.1016/j.ynpai.2022.100105. PMC10039393 36974065

[13] Nisoli E. , Mitochondrial Biogenesis in Mammals: The Role of Endogenous Nitric Oxide, Science. (2003) 299, no. 5608, 896–899. 12574632 10.1126/science.1079368

[14] Melo-Carrillo A. and Lopez-Avila A. , A Chronic Animal Model of Migraine, Induced by Repeated Meningeal Nociception, Characterized by a Behavioral and Pharmacological Approach, Cephalalgia. (2013) 33, no. 13, 1096–1105, 10.1177/0333102413486320, 2-s2.0-84882790131. 23666930

[15] Ashina M. , Migraine and the Trigeminovascular System-40 Years and Counting, Lancet Neurology. (2019) 18, no. 8, 795–804. 31160203 10.1016/S1474-4422(19)30185-1 PMC7164539

[16] Jiang L. , Zhang Y. , Jing F. et al., P2X7R-Mediated Autophagic Impairment Contributes to Central Sensitization in a Chronic Migraine Model with Recurrent Nitroglycerin Stimulation in Mice, Journal of Neuroinflammation. (2021) 18, no. 1, 10.1186/s12974-020-02056-0. PMC7786980 33402188

[17] Demartini C. , Greco R. , Francavilla M. , Zanaboni A. M. , and Tassorelli C. , Modelling Migraine-Related Features in the Nitroglycerin Animal Model: Trigeminal Hyperalgesia is Associated With Affective Status and Motor Behavior, Physiology & Behavior. (2022) 256, 10.1016/j.physbeh.2022.113956. 36055415

[18] Gonzalez-Cano R. , Boivin B. , Bullock D. , Cornelissen L. , Andrews N. , and Costigan M. , Up-Down Reader: An Open Source Program for Efficiently Processing 5.0% Von Frey Thresholds, Frontiers in Pharmacology. (2018) 9, 10.3389/fphar.2018.00433, 2-s2.0-85046639375. PMC5938897 29765323

[19] Christensen S. L. , Hansen R. B. , Storm M. A. et al., Von Frey Testing Revisited: Provision of an Online Algorithm for Improved Accuracy of 50% Thresholds, European Journal of Pain (United Kingdom). (2020) 24, no. 4, 783–790, 10.1002/ejp.1528. 31889375

[20] Foudah A. I. , Devi S. , Alqarni M. H. et al., Quercetin Attenuates Nitroglycerin-Induced Migraine Headaches by Inhibiting Oxidative Stress and Inflammatory Mediators, Nutrients. (2022) 14, no. 22, 10.3390/nu14224871. PMC9695045 36432556

[21] Mao G. X. , Salidroside Protects Human Fibroblast Cells From Premature Senescence Induced by H(2)O(2) Partly Through Modulating Oxidative Status, Mechanisms of Ageing and Development. (2010) 131, no. 11-12, 723–731. 21035481 10.1016/j.mad.2010.10.003

[22] Mao G.-X. , Zheng L. D. , Cao Y. B. et al., Antiaging Effect of Pine Pollen in Human Diploid Fibroblasts and in a Mouse Model Induced by D-Galactose, Oxidative Medicine and Cellular Longevity. (2012) 2012, 750963–10, 10.1155/2012/750963, 2-s2.0-84861017461. 22577492 PMC3345248

[23] Averill-Bates D. A. , The Antioxidant Glutathione, Vitamins & Hormones. (2023) 121, 109–141, 10.1016/bs.vh.2022.09.002. 36707132

[24] Zhang J. Q. , 3-Nitropropionic Acid Induces Ovarian Oxidative Stress and Impairs Follicle in Mouse, PLoS One. (2014) 9, no. 2. 10.1371/journal.pone.0086589 PMC3914797 24505260

[25] Paxinos G. and Watson C. , The Rat Brain in Stereotaxic Coordinates: Hard Cover Edition, 2006, Elsevier.

[26] Nodesa R. and Pain B. R. J. , Migraine pathophysiology: Anatomy of the Trigeminovascular Pathway and Associated Neurological Symptoms, CSD, Sensitization and Modulation of Pain, Pain. (2013) 154, no. Suppl 1. 10.1016/j.pain.2013.07.021 PMC3858400 23891892

[27] Jiménez-Jiménez F. J. , Alonso-Navarro H. , García-Martín E. , Espada-Rubio S. , and Agúndez J. A. G. , Oxidative Stress and Migraine, Molecular Neurobiology. (2024) 61, no. 10, 8344–8360, 10.1007/s12035-024-04114-7. 38499906

[28] Yang F. , Liao J. , Yu W. et al., Exposure to Copper Induces Mitochondria-Mediated Apoptosis by Inhibiting Mitophagy and the PINK1/Parkin Pathway in Chicken (Gallus Gallus) Livers, Journal of Hazardous Materials. (2021) 408, 10.1016/j.jhazmat.2020.124888. 33360697

[29] Gross E. C. , The Metabolic Face of Migraine—From Pathophysiology to Treatment, Nature Reviews Neurology, 2019, Nature Publishing Group, London, UK, 627–643. 10.1038/s41582-019-0255-4 31586135

[30] Xu S. Y. , Song M. M. , Li L. , and Li C. X. , Adie’s Pupil: A Diagnostic Challenge for the Physician, Medical Science Monitor. (2022) 28, 10.12659/msm.934657. PMC8917782 35304432

[31] Chen Z. , Wu H. , Yang J. et al., Activating Parkin-Dependent Mitophagy Alleviates Oxidative Stress, Apoptosis, and Promotes Random-Pattern Skin Flaps Survival, Communications Biology. (2022) 5, no. 1, 10.1038/s42003-022-03556-w. PMC9217959 35732814

[32] Waters C. S. , Angenent S. B. , Altschuler S. J. , and Wu L. F. , A PINK1 Input Threshold Arises From Positive Feedback in the PINK1/Parkin Mitophagy Decision Circuit, Cell Reports. (2023) 42, no. 10, 10.1016/j.celrep.2023.113260. PMC10668033 37851575

[33] Li X. Y. , Yang C. H. , Lv J. J. et al., Global, Regional, and National Epidemiology of Migraine and Tension-Type Headache in Youths and Young Adults Aged 15-39 Years From 1990–2019: Findings From the Global Burden of Disease Study 2019, Journal of Headache and Pain. (2023) 24, no. 1, 10.1186/s10194-023-01659-1. PMC10664527 37993785

[34] Liu L. , Li W. , Wang L. et al., Proteomic and Metabolomic Profiling of Acupuncture for Migraine Reveals a Correlative Link via Energy Metabolism, Frontiers in Neuroscience. (2022) 16, 10.3389/fnins.2022.1013328. PMC9557737 36248663

[35] Gross E. C. , Putananickal N. , Orsini A. L. , Schoenen J. , Fischer D. , and Soto-Mota A. , Defining Metabolic Migraine With a Distinct Subgroup of Patients With Suboptimal Inflammatory and Metabolic Markers, Scientific Reports. (2023) 13, no. 1, 10.1038/s41598-023-28499-y. PMC9992685 36882474

[36] Togha M. , Razeghi Jahromi S. , Ghorbani Z. , Ghaemi A. , and Rafiee P. , An Investigation of Oxidant/Antioxidant Balance in Patients With Migraine: A Case-Control Study, BMC Neurology. (2019) 19, no. 1, 10.1186/s12883-019-1555-4. PMC6911287 31837702

[37] Lisicki M. , D’Ostilio K. , Coppola G. et al., Evidence of an Increased Neuronal Activation-to-Resting Glucose Uptake Ratio in the Visual Cortex of Migraine Patients: A Study Comparing 18FDG-PET and Visual Evoked Potentials, Journal of Headache and Pain. (2018) 19, no. 1, 10.1186/s10194-018-0877-8, 2-s2.0-85049602394. PMC6033847 29978429

[38] Casili G. , Lanza M. , Filippone A. et al., Dimethyl Fumarate Alleviates the Nitroglycerin (NTG)-Induced Migraine in Mice, Journal of Neuroinflammation. (2020) 17, no. 1, 10.1186/s12974-020-01736-1. PMC7469611 32066464

[39] Li J. , Ye X. , Zhou Y. et al., Energy Metabolic Disorder of Astrocytes may be an Inducer of Migraine Attack, Brain Sciences. (2022) 12, no. 7, 10.3390/brainsci12070844. PMC9312932 35884650

[40] Fried N. T. , Moffat C. , Seifert E. L. , and Oshinsky M. L. , Functional Mitochondrial Analysis in Acute Brain Sections From Adult Rats Reveals Mitochondrial Dysfunction in a Rat Model of Migraine, American Journal of Physiology: Cell Physiology. (2014) 307, no. 11, C1017–C1030, 10.1152/ajpcell.00332.2013, 2-s2.0-84914140134. 25252946 PMC4254950

[41] Niu Y. , Zeng X. , Qin G. , Zhang D. , Zhou J. , and Chen L. , Downregulation of Metabotropic Glutamate Receptor 5 Alleviates Central Sensitization by Activating Autophagy via Inhibiting mTOR Pathway in a Rat Model of Chronic Migraine, Neuroscience Letters. (2021) 743, 10.1016/j.neulet.2020.135552. 33352285

